# Differences in Endoscopy Characteristics Between Providers With the Highest and Lowest Post Endoscopy Upper Gastrointestinal Cancer Rates in England

**DOI:** 10.1002/ueg2.70206

**Published:** 2026-04-02

**Authors:** Umair Kamran, Felicity Evison, Eva J. A. Morris, Matthew Brookes, Matt Rutter, David Beaton, Mimi McCord, Nicola J. Adderley, Nigel Trudgill

**Affiliations:** ^1^ Department of Gastroenterology Newcastle Upon Tyne Hospitals NHS Foundation Trust Newcastle UK; ^2^ Data Science Research Development and Innovation University Hospitals Birmingham NHS Foundation Trust Birmingham UK; ^3^ Big Data Institute Nuffield Department of Population Health University of Oxford Oxford UK; ^4^ The Royal Wolverhampton NHS Trust Wolverhampton UK; ^5^ Faculty of Science and Engineering Research Institute in Healthcare Science University of Wolverhampton Wolverhampton UK; ^6^ Department of Gastroenterology North Tees and Hartlepool NHS Foundation Trust Stockton‐on‐Tees and Hartlepool UK; ^7^ Population Health Sciences Institute Faculty of Medical Sciences Newcastle University Newcastle UK; ^8^ Wansbeck Hospital Northumbria NHS Foundation Trust Newcastle upon Tyne UK; ^9^ Heartburn Cancer Basingstoke UK; ^10^ Institute of Applied Health Research University of Birmingham Birmingham UK; ^11^ National Institute for Health and Care Research (NIHR) Birmingham Biomedical Research Centre Birmingham UK; ^12^ Department of Cancer and Genomic Sciences University of Birmingham Birmingham UK; ^13^ Department of Gastroenterology Sandwell and West Birmingham NHS Trust Birmingham UK

**Keywords:** endoscopy, endoscopy volume, gastric cancer, oesophageal cancer, sedation

## Abstract

**Background:**

Post‐endoscopy upper gastrointestinal cancer (PEUGIC) rates vary over threefold between endoscopy providers in England. To determine if variations in endoscopy characteristics contribute, providers with the lowest and highest PEUGIC rates were compared.

**Methods:**

Endoscopy providers were categorized into quartiles based on PEUGIC rates and those in the highest and lowest quartiles studied. Data for diagnostic upper gastrointestinal (UGI) endoscopy performed between January 2019 and February 2020 were extracted from the National Endoscopy Database. Multivariable regression analysis explored the endoscopy characteristics associated with the lowest PEUGIC rate providers after adjusting for patient characteristics and indications.

**Results:**

In total, 328,354 diagnostic UGI endoscopy performed by 54 providers were included. Endoscopy characteristics positively associated with the lowest PEUGIC rate providers included: training sessions (Odds Ratio 1.85 (95% CI 1.81–1.90)); intravenous sedation use (1.09 (1.07–1.11)); endoscopist average UGI endoscopy annual volume 101–200 (1.05 (1.02–1.07) and 201–300 (1.16 (1.13–1.19)). Endoscopy characteristics inversely associated with the lowest PEUGIC rate providers included: endoscopy half‐day sessions with average ≥ 9 points (0.72 (0.71–0.74)); endoscopists not on nursing, specialty or trainee register (0.83 (0.81–0.85)); and biopsies during endoscopy (0.84 (0.83–0.86)). Compliance with national quality standards to biopsy high risk conditions was better in providers with the lowest PEUGIC rates.

**Discussion:**

Training sessions, more endoscopists with minimum annual endoscopy volumes > 100, more intravenous sedation, less biopsies and lower intensity endoscopy sessions were associated with the lowest PEUGIC rate providers. These findings may help guide efforts to reduce PEUGIC and improve endoscopy quality in the future.

## Key Summary

- Summarize the established knowledge on this subject.
  ◦ Post endoscopy upper gastrointestinal cancer (PEUGIC), or cancer potentially missed at endoscopy, is recommended as a key performance indicator for endoscopy providers.
  ◦ PEUGIC rates vary over threefold between endoscopy providers in England.
    However, the potential causes of this variation between endoscopy providers have not been established, limiting quality improvement efforts to reduce the rate of missed cancer at endoscopy.
- What are the significant and/or new findings of this study?
  ◦ In the English National Endoscopy Database analysis, training sessions, more endoscopists with minimum annual endoscopy volumes > 100, more intravenous sedation, less biopsies and more lower intensity endoscopy half‐day sessions were associated with the lowest PEUGIC rate providers.
  ◦ These findings may help guide efforts to reduce PEUGIC and improve endoscopy quality in the future.

## 1 Introduction

In the UK each year, approximately 16,500 people are diagnosed with upper gastrointestinal (UGI) cancer, principally esophageal and gastric cancer, but only one in five survive for more than five years, mainly due to advanced cancer stage at diagnosis. [1] Endoscopy is the investigation of choice to detect UGI cancer. However, studies have reported that 6%–11% of people with UGI cancer had an endoscopy without a cancer diagnosis within the 3 years prior to their UGI cancer being found, representing a potential missed opportunity to detect the cancer earlier. [2, 3, 4, 5, 6] This is termed post‐endoscopy upper gastrointestinal cancer (PEUGIC) and is considered an important key performance indicator of the quality of endoscopy.

A recent population‐based study in England has reported substantial unwarranted variation in PEUGIC rates among endoscopy providers. [7] This variation is likely to be due to differences in the quality of UGI endoscopy between providers. However, it was not possible to explore differences in endoscopy characteristics between endoscopy providers from the administrative and cancer registry databases utilized in this study. The National Endoscopy Database (NED) is a novel UK endoscopy registry overseen by the Joint Advisory Group on Gastrointestinal endoscopy (JAG). [8] It covers > 95% of endoscopy providers in the UK and captures anonymized patient‐level data automatically and in real‐time. This database provides a unique opportunity to examine variations in endoscopy characteristics among providers and to identify quality standards.

We aimed to examine differences in endoscopy characteristics in providers with the highest and lowest PEUGIC rates in England using NED to identify factors which can potentially be addressed to improve endoscopy quality.

## 2 Materials and Methods

### 2.1 Post Endoscopy Upper Gastrointestinal Cancer Rates of Endoscopy Providers

To calculate PEUGIC rates and performance quartiles for the present study, we used the most recent 4 years of data, between 2015 and 2018, from a previous study in which PEUGIC rates among endoscopy providers in England were estimated. [7] The terminology of “endoscopy providers” refers to the organizations which offer NHS endoscopy services in England and an individual organization can have multiple sites. The National Cancer Registration and Analysis Service and Hospital Episode Statistics databases were used to calculate PEUGIC rates for all NHS funded endoscopy providers in England. The total number of patients who were diagnosed with UGI cancers between 2015 and 2018 were 39,959 with overall PEUGIC rate of 8.6% (IQR 7.9–9.5)%.

**FIGURE 1:**
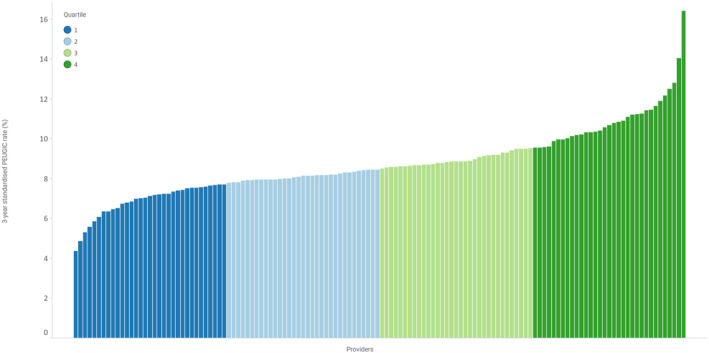
Distribution of PEUGIC rates among endoscopy providers in England. Bars are color coded according to quartiles of provider‐level PEUGIC rates. PEUGIC = Post endoscopy upper gastrointestinal cancer.

PEUGIC rates were adjusted for patient characteristics (age, sex, ethnicity, socioeconomic status, Charlson comorbidity score, a previous diagnosis of esophageal ulcer, esophageal stricture, Barrett's esophagus, gastric ulcer or gastric atrophy), tumor characteristics (tumor histology and site) and endoscopy provider characteristics (accreditation status with the JAG, annual endoscopy volume and provider type (NHS or independent)). Sixty‐four percent of PEUGIC were esophageal cancers and 36% were gastric cancers. Endoscopy providers were categorized into quartiles based on their adjusted PEUGIC rates (Figure 1). The purpose of this categorization was to allow sufficient providers in each group to detect differences in endoscopy characteristics and to reduce the chances of results being influenced by the extremes. Providers in the quartile with the highest PEUGIC rates (median 10.3% (IQR 10.1%–11.4%)) and providers in the quartile with the lowest PEUGIC rates (7.2% (7.0–7.5)%) were included in this study to allow comparison between providers with the highest variability.

### 2.2 National Endoscopy Database

Data for all UGI endoscopy performed in England between January 2019 and February 2020 were obtained from NED. Data were prospectively captured in real time in the NED and analyzed retrospectively. Diagnostic endoscopy performed to investigate UGI symptoms or abnormal findings on previous imaging and for the surveillance of pre‐malignant conditions (such as Barrett's esophagus, gastric atrophy or intestinal metaplasia) or follow up of cancer associated conditions (such as severe esophagitis, esophageal stricture and esophageal or gastric ulcers) were included. Endoscopy were excluded if a therapeutic procedure was performed during endoscopy. Data for UGI endoscopy were mapped to the previously identified endoscopy providers with the lowest and highest PEUGIC rates.

### 2.3 Variables Examined

Variables included information on patient demographics (age and sex), indications for endoscopy categorized into four groups: alarm symptoms (dysphagia, weight loss and anemia), non‐alarm symptoms (dyspepsia, abdominal pain, nausea or vomiting, reflux symptoms, and duodenal biopsies for positive tissue transglutaminase antibodies), abnormal findings on barium or CT scan, and surveillance or follow up. Data on the use of intravenous sedation during endoscopy included the following variables: Midazolam; Fentanyl; Pethidine; Propofol; and General Anesthesia. Data for the endoscopy procedures where combined intravenous sedation (i.e., Midazolam + opioid) was also extracted. Procedures were identified when biopsies were taken during endoscopy, except those endoscopies where only a gastric biopsy was taken for rapid urease testing for the detection of Helicobacter pylori. Data were captured on the number of endoscopic procedures where esophageal intubation could not be achieved.

In the NED, endoscopy half‐day sessions were categorized as service or training sessions. Training sessions were those when a trainee doctor or trainee nurse (clinical) endoscopist was present. Service sessions were those when no trainee was present. In the UK, endoscopy providers use a points system to allocate time for procedures. Usually 15 min are assigned to one point and an UGI endoscopy or flexible sigmoidoscopy is allocated one point and a colonoscopy is allocated two points. [9] Data for all endoscopy (including colonoscopy and flexible sigmoidoscopies, as many endoscopy half‐day sessions included both upper and lower GI procedures) performed by the providers included in the study were also extracted from NED to estimate average workload for half‐day endoscopy sessions for individual providers. We estimated average points allocated to the endoscopy half‐day sessions by an individual provider by calculating the total number of points performed over the study period by the provider and dividing them by the number of half‐day sessions. Average points per half‐day endoscopy session were categorized into < 9 and ≥ 9 points.

The specialities and grade or roles of endoscopists were identified using their General Medical Council (GMC) or Nursing and Midwifery Council (NMC) numbers and categorized into five groups: gastroenterologists (including trainees and consultants); surgeons (including trainees and consultants); nurse endoscopists; others (doctors who are not on specialist or training registers); and unknown if GMC or NMC numbers were not available. In the UK, trainees are always supervised by consultants until they achieve JAG accreditation. Once they achieve accreditation, trainees can perform independent endoscopy. In this study, consultants and trainees were grouped together according to their registration as either “gastroenterologists” or “surgeons”. Nurse endoscopists also need to achieve JAG accreditation in the UK, following which they can work as independent endoscopists. In addition to these, “other” endoscopists were those not registered with the GMC as either consultants or trainees or with the NMC as nurses during the study period. These endoscopists are usually middle grade doctors who have gone through JAG accreditation in UGI endoscopy but did not undergo formal gastroenterological or surgical training to become independent hospital specialists.

The total number of UGI endoscopies performed by each endoscopist over the study period was calculated to estimate their annual UGI endoscopy volume. The British Society of Gastroenterology (BSG) recommends a minimum annual UGI endoscopy volume of 100. [10] In order to identify the most appropriate threshold, endoscopists were categorized into the following groups based on their average annual UGI endoscopy volume: < 100, 101–200, 201–300 and > 300.

### 2.4 Outcome Measures

The association of the above procedural and endoscopist related characteristics at the provider level with the lowest PEUGIC rate provider quartile was examined. Compliance with the BSG endoscopy quality standards [10] was assessed by comparing the proportion of endoscopy in which at least one biopsy was taken from cancer associated conditions (including esophageal ulcer, esophageal stricture and gastric ulcer) and at least six biopsies were taken from a suspected esophageal or gastric cancer.

### 2.5 Statistical Analysis

All statistical analyses were carried out using Stata SE v15 (StataCorp, College Station, Texas, USA). Categorical variables were summarized as number and percentages and the chi‐squared test was used for comparison. Continuous variables were summarized as median and inter quartile range (IQR).

Univariate and multivariate logistic regression modeling was used to explore the characteristics associated with the lowest PEUGIC rate provider quartile. Based on previous studies, [11] clinical experience and availability and accuracy of the data for all variables included in the model were determined a priori. Patient demographics and indications for endoscopy were considered non‐modifiable factors and varied based on the geography of the providers. The purpose of the model was to identify potentially modifiable endoscopy characteristics after adjusting for the variations in non‐modifiable factors. Age was used as a continuous variable. Other categorical variables included sex, indications for endoscopy (alarm symptoms or not), intravenous sedation use, biopsy taken during endoscopy, endoscopist specialty, endoscopist annual volume, and average points per half‐day session per endoscopy provider. All variables were used as procedure level except average points per half‐day session, which was used as a provider level variable.

Multivariable logistic regression analyses were performed to assess associations between endoscopies when at least one biopsy was taken and the providers with lowest PEUGIC rates in the subgroup of endoscopies where a cancer associated condition (esophageal ulcer, esophageal stricture or gastric ulcer) was reported during endoscopy. These models were adjusted for patient sex and age.

### 2.6 Patient and Public Involvement

Patient and public groups, including members from the charity Heartburn Cancer UK and Upper GI Blues, were involved in the study design. A patient and public representative (MM) was also part of the study steering group.

### 2.7 Ethics and Registration

The study protocol was approved by the London ‐ South East Research Ethics Committee (IRAS project ID # 289695). The study was registered with the International Standard Randomised Controlled Trial Number Registry (Registration # ISRCTN44918181).

## 3 Results

### 3.1 Endoscopies Included in the Study

**FIGURE 2:**
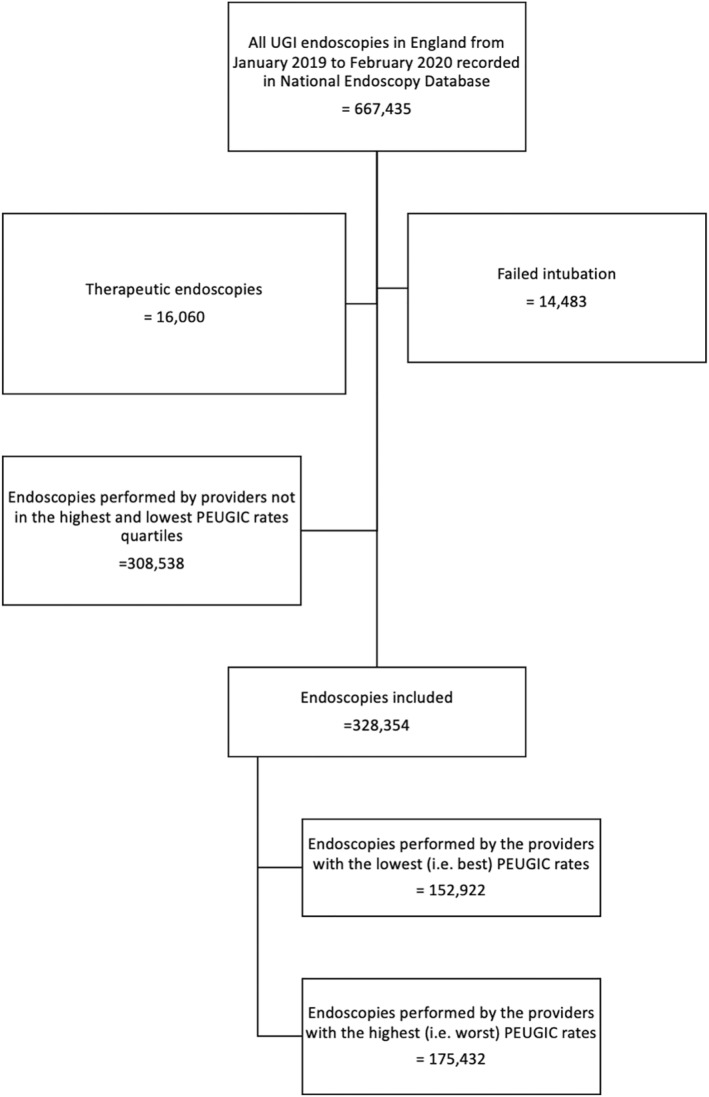
Study flow chart. PEUGIC = Post endoscopy upper gastrointestinal cancer, UGI = Upper gastrointestinal.

A total of 667,435 UGI endoscopies performed by 108 endoscopy providers were recorded in the NED within the study period. Data for 328,354 diagnostic UGI endoscopies performed by 54 endoscopy providers were included: 175,432 endoscopies performed by the 27 providers with the highest PEUGIC rates (median 10.3% (10.1–11.4)) and 152,922 endoscopies performed by the 27 providers with the lowest PEUGIC rates (7.2% (7.0–7.5)) (Figure 2). These endoscopies were performed by 2365 endoscopists in total, 1295 endoscopists working for the providers with the lowest PEUGIC rates and 1070 endoscopists working for the providers with the highest PEUGIC rates. Failed intubation was more common in providers with the highest PEUGIC rates (1.9%) compared with providers with the lowest PEUGIC rate (1.6%), *p* < 0.001. Endoscopy was more commonly performed for female patients and the median patient age was 61 (IQR 47–62) years. Non‐alarm symptoms were the commonest indications followed by alarm symptoms and surveillance or follow up endoscopy (Table 1).

**TABLE 1:** Demographics of patients and characteristics of endoscopies stratified by the quartiles of providers with the highest and lowest post endoscopy upper gastrointestinal cancer rates.

Variables	Providers with the highest PEUGIC rates	Providers with the lowest PEUGIC rates
PEUGIC rates^a^ Median (IQR), %	10.3 (10.1–11.4)	7.2 (7.0–7.5)
Endoscopies included	175,432	152,922
Patient age (years)		
Less than 40	26,068 (14.9%)	22,832 (14.9%)
41–50	23,234 (13.2%)	20,188 (13.2%)
51–60	33,766 (19.2%)	29,159 (19.2%)
61–70	35,444 (20.2%)	30,382 (19.9%)
71–80	36,084 (20.6%)	31,440 (20.6%)
> 80	19,751 (11.3%)	18,029 (11.8%)
Female patients	93,532 (53.3%)	79,802 (52.2%)
Indications		
Non‐alarm symptoms	128,587 (73.3%)	114,122 (74.6%)
Abnormal imaging	2247 (1.3%)	1757 (1.1%)
Surveillance or follow up	11,163 (6.4%)	9123 (6.0%)
Alarm symptoms	33,435 (19.1%)	27,920 (18.3%)
Training session	14,685 (8.4%)	22,261 (14.6%)
Use of intravenous sedation	91,869 (52.4%)	81,220 (53.1%)
Use of combined intravenous sedation^b^	22,866 (13.0%)	23,318 (15.2%)
Biopsy taken^c^	79,709 (45.4%)	64,150 (42.0%)
Endoscopist specialty		
Gastroenterologist	90,919 (51.8%)	77,115 (50.4%)
Nurse endoscopist	28,026 (16.0%)	31,383 (20.5%)
Surgeon	32,192 (18.4%)	27,245 (17.8%)
Other doctors (not on specialist or trainee registers)	23,757 (13.5%)	15,947 (10.4%)
Unknown	538 (0.3%)	1232 (0.8%)
Annual endoscopist volume		
< 100	27,157 (15.6%)	24,277 (15.9%)
101–200	37,082 (21.3%)	32,775 (21.4%)
201–300	29,340 (16.8%)	25,831 (16.9%)
> 300	80,793 (46.3%)	69,233 (45.3%)
Average points per half‐day endoscopy session		
< 9	143,878 (82.0%)	132,298 (86.5%)
≥ 9	31,554 (18.0%)	20,624 (13.5%)

*Note:* Values are numbers (percentages) unless stated otherwise.

Abbreviation: PEUGIC = Post endoscopy upper gastrointestinal cancer.

^a^ PEUGIC rates calculated using data between 2015 and 2018 extracted from our previous study [5].

^b^ Combined use of benzodiazepines and opioids.

^c^ At least one biopsy taken (except for rapid urease test).

UGI cancer was suspected in 1% of endoscopies. Gastric ulcers were reported in 1.9%, esophageal ulcers in 1.2% and esophageal strictures in 0.6% of endoscopies.

Overall, 11.3% of endoscopies were performed during a training session and intravenous sedation was used for 52.7% of the procedures. The majority (69.3%) of the procedures were performed by endoscopists registered as specialists (consultants medical or surgical) or specialty trainee doctors. Nurse endoscopists performed 18.1% of endoscopies, and 12.1% were performed by the endoscopists that were neither specialists nor trainees.

### 3.2 Endoscopy Characteristics Associated With the Lowest PEUGIC Rate Providers

In multivariable regression analysis after adjusting for patient age, sex, and indications for the procedure, the endoscopy characteristics positively associated with the providers with the lowest PEUGIC rates included: intravenous sedation use, endoscopy performed during training sessions, endoscopists' average annual UGI endoscopy volume, and endoscopy performed by nurse endoscopists. Endoscopy characteristics inversely associated with providers with the lowest PEUGIC rates included: endoscopy half‐day sessions with average ≥ 9 points; endoscopy performed by other doctors not on specialist or trainee registers, and biopsies during endoscopy (Table 2). Combined use of intravenous benzodiazepines and opioids was more common among the providers with lowest PEUGIC rates (15.2% vs. 13.0%, *p* < 0.001).

**TABLE 2:** Univariate and multivariate regression analysis of factors associated with providers with the lowest post endoscopy upper gastrointestinal cancer rates.

Factors	Univariable analysis OR (95% CI)	Multivariable analysis OR (95% CI)
Sex of the patient		
Female	Reference	
Male	1.00 (0.98–1.02)	1.01 (0.99–1.02)
Age of the patient (years)		
< 40	Reference	
41–50	0.99 (0.97–1.02)	1.01 (0.98–1.04)
51–60	0.98 (0.96–1.01)	1.01 (0.99–1.03)
61–70	0.98 (0.96–1.00)	1.01 (0.98–1.03)
71–80	0.99 (0.97–1.02)	1.04 (1.02–1.07)
> 80	1.04 (1.01–1.07)	1.11 (1.08–1.14)
Indications for endoscopy		
Non‐alarm symptoms	Reference	
Abnormal CT scan	0.88 (0.83–0.94)	0.90 (0.84–0.96)
Surveillance or follow up	0.92 (0.89–0.95)	0.92 (0.90–0.95)
Alarm symptoms	0.94 (0.92–0.96)	0.95 (0.93–0.98)
Endoscopy session type		
Service	Reference	
Training^a^	1.86 (1.82–1.91)	1.85 (1.81–1.90)
Intravenous sedation used		
No	Reference	
Yes^a^	1.03 (1.02–1.04)	1.09 (1.07–1.11)
Biopsies taken		
No	Reference	
Yes^a^	0.87 (0.86–0.88)	0.84 (0.83–0.86)
Endoscopist specialty		
Gastroenterologist	Reference	
Nurse endoscopist^a^	1.32 (1.30–1.35)	1.45 (1.42–1.48)
Surgeon	0.99 (0.98–1.01)	1.00 (0.98–1.02)
Other doctors (not on specialist or trainee registers)^a^	0.79 (0.77–0.81)	0.83 (0.81–0.85)
Annual endoscopist volume		
< 100	Reference	
101–200^a^	0.99 (0.97–1.01)	1.05 (1.02–1.07)
201–300^a^	0.98 (0.96–1.01)	1.16 (1.13–1.19)
> 300^a^	0.96 (0.94–0.98)	1.09 (1.06–1.11)
Average points per half day endoscopy session		
< 9	Reference	
≥ 9^a^	0.71 (0.70–0.72)	0.72 (0.71–0.74)

*Note:* OR > 1 indicates a higher odds ratio of being in the quartile with lowest PEUGIC rates compared to the areference category; OR < 1 indicates a lower odds ratio.

^a^ Denotes statistically significant results.

### 3.3 Compliance With Quality Standards to Take Biopsies From High‐Risk Conditions

Although the overall biopsy rate was lower in the lowest PEUGIC rate providers (41.4% vs 44.5%), biopsy rates were higher if a high risk finding was recorded in the endoscopy report compared to the providers with highest PEUGIC rates: esophageal ulcers — lowest PEUGIC rate providers 56.7% versus highest PEUGIC rate providers 51.3% (*p* = 0.001); esophageal strictures — 52.4% versus 48.0% (*p* = 0.042); and gastric ulcers ‐ 62.7% versus 58.3% (*p* < 0.001). In multivariable regression analyses, biopsies for these high‐risk conditions were associated with the lowest PEUGIC rate provider quartile (Table 3).

**TABLE 3:** Multivariable analysis of the association between biopsy rates for esophageal ulcers, esophageal strictures, and gastric ulcers and providers with the lowest post endoscopy upper gastrointestinal cancer rates adjusted for patient age and sex (compared to endoscopy with no biopsies).

Subgroups	Number of patients *N*	Biopsy rate *N* (%)	Association with providers with the lowest PEUGIC rates Odds ratio (95% confidence interval)
Esophageal ulcer	3859	2090 (54.2)	1.23 (1.08–1.40)
Esophageal stricture	2070	1036 (50.1)	1.19 (1.00–1.42)
Gastric ulcer	6270	3792 (60.5)	1.20 (1.09–1.33)

*Note:* OR > 1 indicates a higher odds ratio of being in the quartile with lowest PEUGIC rates compared to the reference category; OR < 1 indicates a lower odds ratio.

Abbreviation: PEUGIC = Post endoscopy upper gastrointestinal cancer.

However, there was no significant difference between different PEUGIC rate providers in the proportion of endoscopy when at least six biopsies were taken from suspected UGI cancers (lowest PEUGIC rate 73.9% vs. highest 70.7%, *p* = 0.09).

## 4 Discussion

UGI endoscopy lacks well established quality indicators due to a relative paucity of evidence. PEUGIC has been recommended as an important key performance indicator for endoscopy providers [10] but is limited in its impact as it is a relatively infrequent event particularly for individual endoscopists and necessarily retrospective in its application. This study has reported differences in endoscopy characteristics between providers with the lowest and highest PEUGIC rates in England. These findings can help to reduce variation in endoscopy practice among providers and guide future quality improvement efforts to reduce PEUGIC.

Intravenous sedation is commonly used to improve the tolerability of UGI endoscopy. In England, nearly all UGI endoscopies are performed with local anesthetic throat spray alone or conscious sedation. Most sedated endoscopies are carried out using benzodiazepines, with or without opioids. A recent multicentre study from China reported that propofol sedation improved the detection rate of early UGI cancer and high grade dysplasia. [12] These observations are likely to relate to better patient procedural tolerance with sedation, allowing longer and more effective inspection times and easier use of accessory image enhancement techniques to detect early neoplasia and pre‐malignant conditions. [12, 13] The current and previous studies suggest that where appropriate based on patient factors, intravenous sedation should be offered to all patients undergoing diagnostic or surveillance endoscopy.

Annual endoscopist UGI endoscopy volume has been proposed as a key performance indicator. [10, 14] A recent root cause analysis study of PEUGIC has reported a negative correlation between annual endoscopist volume and the PEUGIC rate. [15] The current study supports the BSG recommendations for minimum annual volumes of more than 100 UGI endoscopies. There was a marginally stronger association with an endoscopist annual volume of 201–300, suggesting that an aspirational standard of more than 200 UGI endoscopies per year would be appropriate. Most UK endoscopists undertake both upper and lower GI endoscopy. A previous NED analysis reported that only around half of the endoscopists in the UK met the 100 UGI endoscopies per year minimum recommended volume. [16] The present study also found a difference in the failed intubation rate, which could also be used as a potential key performance indicator.

A negative association was found between the number of endoscopies performed by endoscopists not on specialist, trainee or nursing registers and the lowest PEUGIC rate providers quartile, which reflects a potential impact of endoscopists' specialty and training on the quality of endoscopy. Endoscopists not on a specialist or training register in England may not participate or have the same opportunities for formal training courses or continuous professional development activities to improve their endoscopy and lesion recognition skills. Studies have shown that training in lesion recognition can improve the diagnostic yield of endoscopy and involvement in such training programs should be encouraged for all endoscopists. [17] Similar results have been reported for colonoscopy, when the involvement of trainees in screening colonoscopy improved adenoma detection rates. [18].

A multicentre cohort study reported that endoscopists with the highest biopsy rates were five times more likely to diagnose gastric cancer and the incidence of the missed gastric cancers was also lower compared with the endoscopists with the lowest biopsy rates. [19] In contrast, the current study found a negative association between overall biopsy rate and the providers with lowest PEUGIC rates. On further analysis, it was noted that compliance with the national recommendations to take biopsies when a high risk cancer associated lesion was identified [10] was higher by providers with the lowest PEUGIC rates. These findings suggest that, although in England overall biopsy rate may not be an indicator of endoscopy quality, compliance with national recommendations to take biopsies when assessing high risk cancer associated lesions is an indicator of high quality UGI endoscopy. It also suggests that although the endoscopists in endoscopy providers with low PEUGIC rates take less biopsies overall, they take more targeted biopsies, which is likely a reflection of their training and confidence in optical diagnosis of lesions. This issue merits further study and is an opportunity for quality improvement.

The present study identified an association between less intense endoscopy half‐day sessions and the lowest PEUGIC rate provider quartile. Currently, there are no benchmarks for how many procedures should be booked in a half‐day endoscopy session. The points allocation system is a widely accepted method for scheduling patients for endoscopy in the UK, that is adjusted for case mix, training, endoscopy session length and process factors such as room turnaround time. Busy endoscopy sessions may result in undue pressure on endoscopists, limiting their ability to spend adequate time examining the UGI mucosa for pre‐malignant and early malignant changes. Intense endoscopy sessions may also contribute to operator fatigue, which can affect the quality of endoscopy. Previous studies on colonoscopy have reported that colonoscopist fatigue is associated with reduced adenoma detection. [20, 21, 22].

The current study has several important limitations. NED data were not exactly contemporaneous with the data which were used to categorize endoscopy providers into quartiles based on PEUGIC rates. It is unlikely that endoscopy characteristics would have significantly changed over this short period of time and affected the results of the study, but this cannot be excluded. Severe reflux esophagitis is not coded in HES, and therefore PEUGIC rates could not be adjusted for this potential risk factor. Data on important factors such as patient comfort, the quality of views during endoscopy, whether any mucosal cleansing and enhanced imaging techniques were used, procedure time, and the experience and training of endoscopists, number of years out of training and endoscopist sex were not available in the current iteration of NED. Data on some of these factors will be available in the second iteration of NED, which has already been launched in the UK and future studies should analyze these measures in relation to PEUGIC rates. Average points per half‐day session were calculated using the points usually allocated to diagnostic procedures. It is possible that some of the endoscopy on these sessions were planned therapeutic procedures and would have been allocated extra points, which could potentially lead to an underestimation of the average points per half‐day session. However, therapeutic procedures form a very small proportion of overall endoscopy and this should not affect the results of this study.

Strengths of this study include the use of a large, prospectively collected, multicentre database, providing robust statistical power, standardized data capture, and good generalizability to routine clinical practice. Comparing endoscopy characteristics between the providers with highest and lowest PEUGIC rates maximizes contrast between performance extremes, facilitating identification of factors associated with variation in outcomes and supporting clinically meaningful interpretation.

## 5 Conclusions

This study has identified important differences in endoscopy characteristics between providers with the highest and lowest PEUGIC rates in England. We therefore suggest that endoscopist sedation and failed intubation rates, endoscopy half‐day session procedure volumes and annual endoscopist UGI endoscopy volumes all merit monitoring and intervention where appropriate. Furthermore, biopsy practice for cancer associated lesions and potentially unnecessary biopsies from minor findings such as gastritis or duodenitis may merit monitoring and intervention.

## Author Contributions

The study concept and design were jointly conceived by U.K., N.J.A. and N.T. Data extraction was performed by F.E. and U.K. Analysis was performed by U.K and N.J.A. The manuscript was drafted by U.K. The manuscript was critically reviewed, revised, and approved by all authors.

## Funding

This project was funded by the National Institute for Health and Care Research (NIHR) under its Research for Patient Benefit (RfPB) Program (Grant Reference Number NIHR 201571). The views expressed are those of the authors and not necessarily those of the NIHR or the Department of Health and Social Care. EM was supported by the NIHR Oxford Biomedical Research Center.

## Conflicts of Interest

The authors declare no conflicts of interest.

## Data Availability

The data that support the findings of this study are not available on request from the corresponding author. The data are not publicly available due to privacy or ethical restrictions. We are grateful to the Joint Advisory Group on GI Endoscopy for access to data from the National Endoscopy Database.
